# A Thematic Review on Using Food Delivery Services during the Pandemic: Insights for the Post-COVID-19 Era

**DOI:** 10.3390/ijerph192215267

**Published:** 2022-11-18

**Authors:** Yezheng Li, Pinyi Yao, Syuhaily Osman, Norzalina Zainudin, Mohamad Fazli Sabri

**Affiliations:** 1 Business School, Guilin University of Technology, Guilin 541004, China; 2 Faculty of Human Ecology, Universiti Putra Malaysia, Serdang 43400, Malaysia

**Keywords:** food delivery app, online food delivery, online-to-offline (O2O), consumer behavior, COVID-19, post-pandemic, thematic review

## Abstract

The food delivery service is the most typical and visible example of online-to-offline (O2O) commerce. More consumers are using food delivery services for various reasons during the COVID-19 pandemic, making this business model viral worldwide. In the post-pandemic era, offering food delivery services will become the new normal for restaurants. Although a growing number of publications have focused on consumer behavior in this issue, no review paper has addressed current research and industry trends. Thus, this paper aims to review the literature published from 2020 to the present (October 2022) on consumers’ use of food delivery services during the pandemic. A thematic review was conducted, with 40 articles searched from Scopus and Web of Science being included. Quantitative findings showed current research trends, and thematic analyses formed eight themes of factors influencing consumer behavior: (1) technical and utilitarian factors, (2) system-related attributes, (3) emotional and hedonic factors, (4) individual characteristics, (5) service quality, (6) risk-related factors, (7) social factors, and (8) food-related attributes. The paper also emphasizes COVID-19-related influences and suggests promising future research directions. The results offer insights into industry practices and starting points for future research.

## 1. Introduction

The rapid expansion of electronic commerce (e-commerce) or mobile commerce (m-commerce) is changing people’s food consumption patterns, with more and more consumers considering purchasing food online. They generally purchase food online in business-to-consumer (B2C) and online-to-offline (O2O) models. B2C is a traditional e-commerce model where consumers purchase food from B2C platforms (e.g., China’s JD.com and USA’s Amazon.com) and receive the parcel in approximately 3–10 days, while O2O is a new e-commerce model focused on local business where consumers order online and then consume offline [1]. In the O2O model, consumers can visit the offline store or use the home delivery service. The former is called to-shop O2O, while the latter is known as to-home O2O [2]. Food delivery is the most obvious and widely discussed O2O market segment, in which restaurants work with third-party O2O platforms, i.e., online food delivery platforms, to offer delivery of ready-to-eat food [3]. Consumers can easily find nearby restaurants through the food delivery app, accessing the convenience and diversity of food delivery services. Although the food delivery market has continued to expand since the emergence of the O2O concept, its growth was uneventful [3] until the outbreak of coronavirus disease 2019 (COVID-19).

COVID-19 is an infectious disease caused by the severe acute respiratory syndrome coronavirus 2 (SARS-CoV-2) [4]. The World Health Organization (WHO) declared its outbreak in January 2020 and subsequently called it a pandemic in March 2020. During the pandemic, the WHO strongly advises that people wear masks in public places, maintain social distancing, self-isolate, and take other self-protective actions to avoid contracting COVID-19 [5,6]. In the early stages of the pandemic, many countries worldwide adopted strict measures such as lockdowns, quarantine, and movement control to reduce the risk of COVID-19 spread. Industries around the world, especially the food and restaurant industries, have been severely impacted as a result because consumers use fewer public services or dine less in public places [7,8].

The COVID-19 pandemic has significantly changed consumer behavior, with more and more people purchasing food online due to social distancing policies or fear of infection. Since food is a daily necessity for individuals, buying food through to-home O2O (i.e., instant food delivery) seems to meet consumer needs better than B2C and is more popular. To sustain the business, many restaurants started to access online platforms offering food delivery services to meet consumers’ demands [7,9]. According to statistics, the COVID-19 pandemic led to unprecedented growth in food delivery services, with restaurant food delivery growing 47% worldwide in 2020; more than 1.6 billion people globally used some form of online food delivery service in 2021 [10,11]. In a way, the COVID-19 pandemic has accelerated the digitalization of numerous industries, including the restaurant industry [6,12,13]. On the other hand, the pandemic disrupted the pre-existing food delivery market, with users more cautious in their decision to continue using the food delivery service due to concerns about the safety of the delivered food [14,15].

Previous studies have investigated the factors influencing consumer behavior in the context of online food delivery service from different perspectives, such as technology adoption [16,17,18], service quality [19,20], food choice motives [1,21], etc. However, research has shown that many aspects of consumer expectations during the pandemic differ from normal times [9] and that consumer purchasing behavior during the crisis is unusual [22]. Consumers’ thinking and behavior are being reshaped by COVID-19 [9,23,24]. This means that new factors may influence consumer behavior, while the identified factors may work in different ways. In fact, many studies have focused on using food delivery services during the COVID-19 pandemic. However, no review paper has attempted to explore the research and industry trends of food delivery in the (post-) pandemic era. Accordingly, this paper aims to identify these trends by reviewing the current literature.

In the post-pandemic era, continued research on consumer behavior regarding food delivery services is necessary for the following reasons: First, consumers will adapt to the new normal situation, and some behavioral changes resulting from the pandemic might continue. Consumer behavior is generally highly predictable; nonetheless, many aspects of the COVID-19 pandemic increase prediction uncertainty [25]. Second, food deliveries will be the new normal for restaurants and diners in the foreseeable future [26,27], and a comprehensive understanding of consumer behavior can help businesses remain resilient in their business. Lastly, food delivery as a segment of O2O commerce is not restricted to delivering ready-to-eat food [2,28]. The combined effect of innovation and COVID-19 has given rise to many new business models. This is an effort by restaurants and food delivery platforms to sustain their business, which may become the new fashion after the pandemic fades. New insights and marketing strategies are hence needed in the post-pandemic era. Therefore, another objective of this paper is to identify literature gaps to offer a starting point for future research.

## 2. Materials and Methods

This paper collected literature from dominant online databases and used a non-systematic approach to review. Most systematic reviews aim to measure the effectiveness of prior studies rigorously and scientifically to reveal whether their findings are consistent across studies [29]. In contrast, non-systematic reviews seek to identify what the literature says about a particular issue and where effective research should be conducted [29]. In this paper, the non-systematic approach was adopted based on the research objectives: identifying research and industry trends regarding food delivery services use in the COVID-19 pandemic and offering future research directions. Furthermore, a systematic review would not be particularly useful or effective if only a limited number of published studies were published in a given field [29]. Since the COVID-19 pandemic began in 2020, it can be expected that there are not many relevant studies. For example, studies involving the relationship between perceived risk from COVID-19 and consumers’ use of food delivery apps might be limited. Hence, the non-systematic review approach was more appropriate for this paper. Specifically, a thematic analysis procedure was used, which is a typical design for non-systematic reviews [30].

However, the non-systematic review does not mean it is not rigorous and scientific. In fact, any non-systematic review must be systematic to some extent to ensure its credibility [29]. Thus, this paper adopted Zairul’s [31,32,33] thematic review method and conducted a thematic review following the steps suggested by Greetham [29].

### 2.1. Formulating the Research Question

The research questions can help a researcher judge what is relevant to their topic, providing clarity, cohesion, and direction to their work. In this paper, we follow the research questions to gather, structure, and analyze the literature in the following steps. Therefore, we proposed the following research questions:

- RQ1: What are the current trends of the food delivery service discussed in the literature related to consumer behavior during the COVID-19 pandemic?
- RQ2: What factors influence consumer behavior in using food delivery services during the COVID-19 pandemic?
- RQ3: What are the characteristics or changes in consumer behavior using food delivery services during the COVID-19 pandemic?

### 2.2. Literature Screening

In order to determine which article should be reviewed, we developed explicit inclusion and exclusion criteria. First, we determined “food delivery” and “COVID-19” as the core keywords after a preliminary study of industry reports and previous literature. The title of the article to be reviewed must simultaneously include these two core keywords or their synonyms. Second, we only selected peer-reviewed journal articles to ensure the quality of the research to be reviewed. However, review articles were excluded because of the contradiction with the objective of our paper. Third, we only considered articles written in English. Fourth, the articles to be included must focus on consumers’ use of food delivery services during the COVID-19 pandemic. Last, studies must involve the influence of the COVID-19 pandemic on consumer behavior rather than simply using it as a writing background.

### 2.3. Searching the Literature

**Figure 1:**
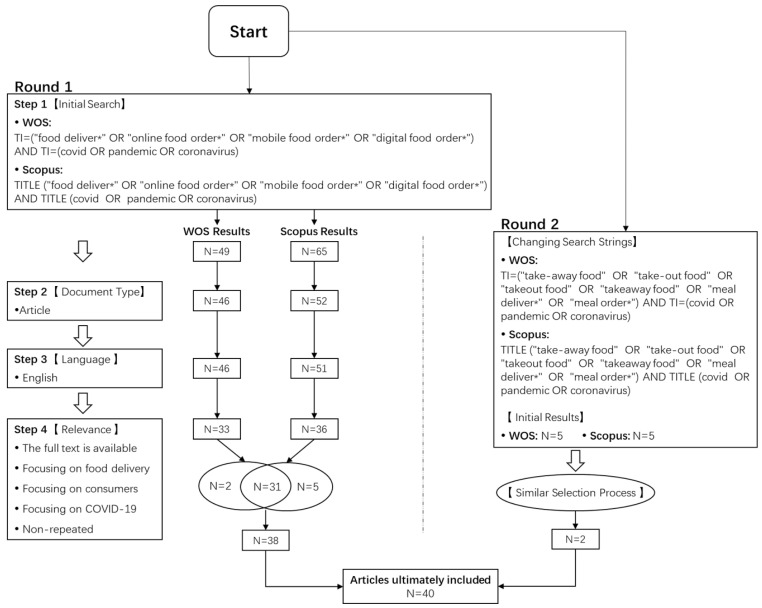
Literature searching process.

We chose two prevailing citation databases, namely the Scopus and the Web of Science (WOS) core collection, from which to search articles. We searched and selected the literature according to the inclusion and exclusion criteria as mentioned previously, with the procedure shown in Figure 1. All authors searched and assessed articles from the two databases separately, following the same procedure. We discussed the respective search results and repeated the above procedure until the results were consistent. Given that “food delivery” may have many synonyms in different contexts, we changed the keywords to search again to avoid missing valuable articles. In addition, those articles with repeated content using the same data were excluded. Ultimately, 40 articles that met the criteria published from 2020 to the present (updated on 15 October 2022) were included in this review, as shown in Table A1 of Appendix A.

### 2.4. Data Extraction and Synthesis

Following Zairul’s [31,32,33] method, we used the ATLAS.ti 9 software (ATLAS.ti GmbH, Berlin, Germany) to extract and synthesize data, importing documents into the software for further thematic review. Specifically, we read the articles thoroughly and performed a thematic analysis procedure to construct the themes, which were identified by an iterative process of comparing similarities and differences between the reviewed subjects to achieve consistency. The bibliometric information and other directly identifiable metadata from the reviewed articles were extracted for descriptive quantitative analysis. Subsequently, we used a coding method similar to the qualitative study to conduct the thematic analysis. We coded the influencing factors of consumer behavior in using food delivery services during the COVID-19 pandemic and categorized them into several themes after several rounds of recoding and merging codes.

## 3. Results and Discussion

The results were classified into quantitative findings and qualitative findings. The former was mainly used to answer RQ1, while the latter addressed RQ2 and RQ3. The discussion in each subsection may involve literature outside of the reviewed articles for illustrative purposes.

### 3.1. Quantitative Results

**Figure 2:**
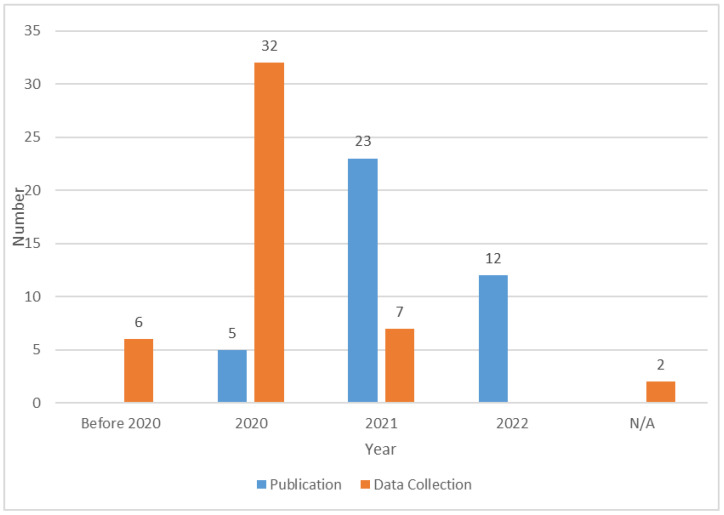
The year of publication and data collection.

Research trends were examined by general bibliometric information and directly identifiable metadata, including the year of publication and data collection, source of publication, study site, and key dependent variables or research focuses. First, as shown in Figure 2, the earliest studies appeared in 2020 because that is when the COVID-19 pandemic began. We checked the time of data collection for each study and marked it as Not Applicable (N/A) when it could not be identified or inferred. Figure 2 shows that most of the studies collected data in 2020, of which six (i.e., [34,35,36,37,38,39]) collected data before and after 2020 for comparative analysis. In addition, Meena and Kumar [9] analyzed what consumers posted online in 2020 and 2021 and showed that consumers’ net sentiment (positive-negative) during the second wave of COVID-19 was significantly higher than that during the first wave, which may be due to consumers psychologically adapting to the new normal. Therefore, consumers’ behavioral characteristics or changes regarding the use of using food delivery services in the post-pandemic era are worth investigating. However, even in the articles published in 2022, few studies used data from 2021 and beyond. One possible reason is that the articles in question have not yet been officially published.

Second, 40 articles were published in 25 different journals, as shown in Table A1 in Appendix A, indicating that consumers using food delivery services during the COVID-19 overhaul is an interdisciplinary and widely discussed issue. Regarding the study site, we were interested in where the study was conducted and where the researcher focused. This is because economic and cultural contexts are important aspects of understanding consumer behavior. As can be seen from Table 1, studies involved 17 countries or regions with different cultures and continents, suggesting that the issue is a globally widespread phenomenon.

**Figure 3:**
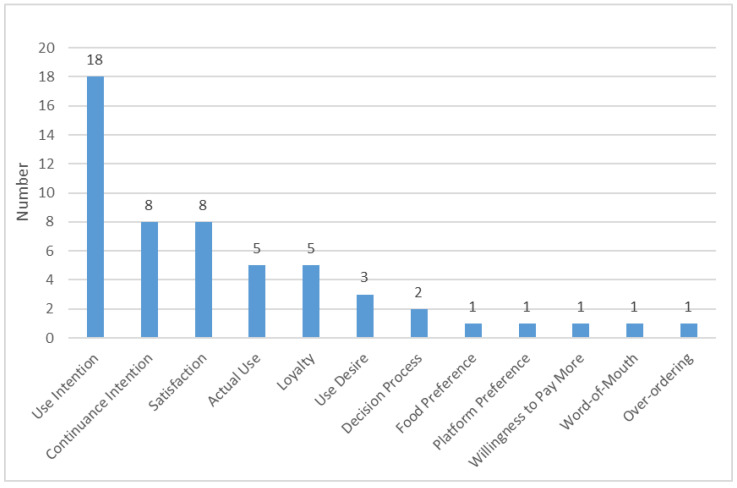
Key dependent variables.

We also examined key dependent variables or research focuses of articles reviewed to identify the scope of consumer behavior and present the research trends. As presented in Figure 3, consumer behavior mainly involved use intention, continuance intention, satisfaction, actual use, loyalty, etc. Some articles involved several keywords and vice versa. In addition, some studies have analyzed online comments [9,24,27,47] or conducted general behavioral surveys [41,45] to understand consumer behavior towards using food delivery services during the pandemic. Previous studies argued that the use of food delivery services may be associated with some outcome phenomena, such as sedentary lifestyles [62] or environmental pollution issues [63]. However, in the articles reviewed, no studies focused on outcome phenomena related to the use of food delivery services, except for Sharma et al. [50] investigating consumers’ over-ordering phenomenon.

In summary, this section answers research question 1, in which research trends reflected the industry trends to some degree.

### 3.2. Qualitative Results

**Figure 4:**
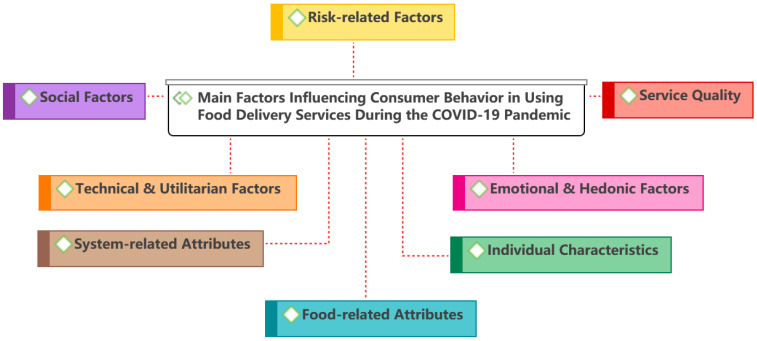
Main factors influencing consumer behavior.

We thoroughly read all the articles and coded consumer behavior and its influencing factors. Several rounds of recoding, merging, and categorization was conducted on the initial codes. Since we are concerned with factors that are broadly considered and validated by researchers, the codes that were not used frequently and could not be categorized as any theme were excluded, as well as non-significant results. Specifically, we first coded the factors that directly or indirectly influence consumer behavior in terms of using food delivery services, generating eight main themes (see Figure 4 and Section 3.2.1). Subsequently, COVID-19-related influences were highlighted (see Section 3.2.2).

#### 3.2.1. General Themes

**Figure 5:**
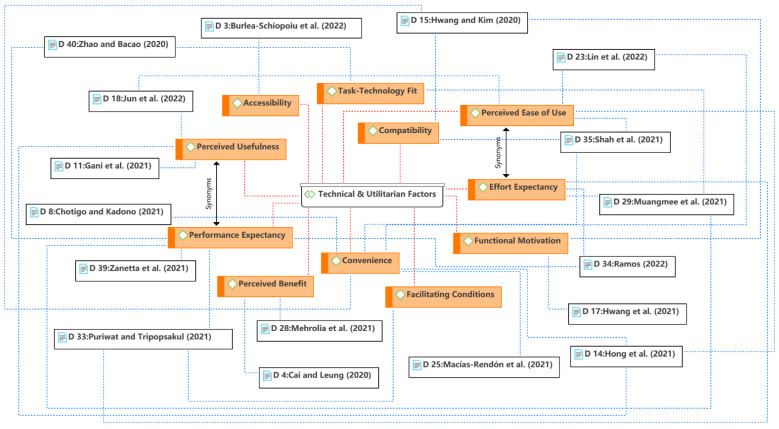
Theme 1: technical and utilitarian factors [6,12,13,15,26,35,36,37,42,44,46,48,53,54,55,56,58].

The first theme is the technical and utilitarian factors, as shown in Figure 5. The popularity of food delivery services cannot be separated from the development of e-commerce and mobile internet. As a result, the discussion always centers on the technological aspect, whether before or during the COVID-19 pandemic. Consistent with the e-commerce literature, social psychology-based technology adoption theories were often used to explain consumer behavior in the context of food delivery services, such as the technology acceptance model (TAM) [64,65] and the unified theory of acceptance and use of technology (UTAUT) [66,67]. Perceived usefulness, perceived ease of use, and their synonyms constituted the main aspects of the theme. This means that consumers are more likely to use a food delivery app if they find it useful and easy to use. Such technical attributes highlight the extrinsic (utilitarian) motivation of consumers’ technology usage. Extrinsic motivation is also expressed in terms of perceived benefits, convenience, functional aspects, etc. In addition, UTAUT suggested that facilitating conditions or compatibility is also a key factor in determining consumer use of a particular technology.

**Figure 6:**
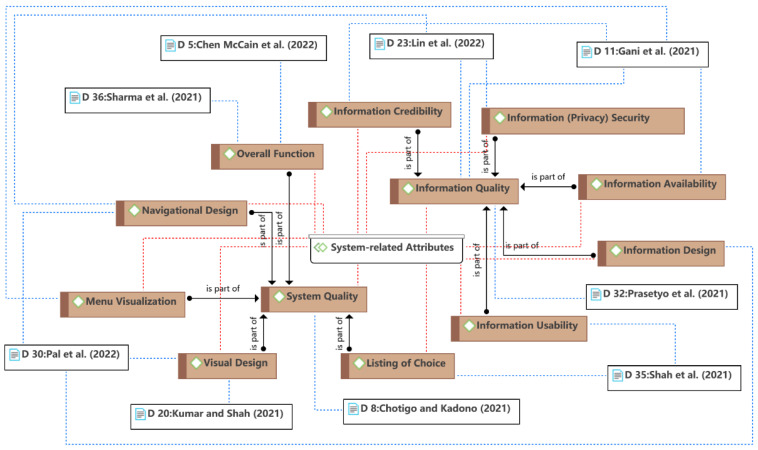
Theme 2: system-related attributes [7,12,27,35,44,46,49,50,51].

The second theme is the system-related attributes, as shown in Figure 6. Consumers use food delivery services typically through mobile apps, so many studies have focused on the influence of the quality of information systems on consumer behavior. System-related attributes focused on the specific functional aspect of the food delivery app. According to the information systems success model (ISSM) [68,69], a successful information system involves three main exogenous factors: system quality, information quality, and service quality. This theme was mainly concerned with the first two factors, while service quality was discussed separately in Theme 5 because it includes not only the online part (i.e., the system aspect) but also the offline part in the context of food delivery. System-related attributes often indirectly influence consumer behavior and decision-making processes. For example, information quality and system quality may contribute to perceived usefulness [12] (see Theme 1); visual design (facility aesthetics) may evoke consumers’ emotions such as dominance and pleasure [7] (see Theme 3).

**Figure 7:**
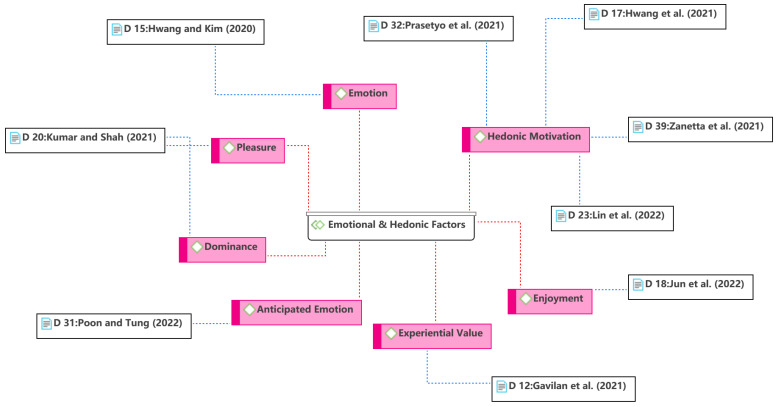
Theme 3: emotional and hedonic factors [7,28,37,42,44,51,52,55,58].

The third theme is the emotional and hedonic factors, as shown in Figure 7. Apart from cognitive-based extrinsic (utilitarian) factors (see Theme 1), emotional-based hedonic motivations are also key factors influencing consumers’ technology use [67]. However, with increasing experience, the novelty contributing to hedonic motivation become less attractive [67]. Furthermore, researchers should be cautious in using hedonic motivation as a predictor in the context of focusing on utilitarian aspects (e.g., food delivery service). Previous research has shown a non-significant result of hedonic motivation [17]. Surprisingly, this review found a number of studies that reported significant effects of hedonic motivation. One possible explanation is that food delivery services may be novel to specific communities, such as new users, or that the study is on a new food delivery business model, such as drone food delivery [37]. On the other hand, the fun may not come from the use of technology but from other aspects. For example, people had limited outdoor recreation during the COVID-19 pandemic, making online ordering a meal potentially fun. The other emotional aspects have also been the focus of many studies. During the pandemic, food delivery services helped restaurants maintain their businesses, provided jobs to the unemployed, and delivered food and medicine to consumers, which enhanced the emotional connection between people and food delivery platforms [7]. In addition, consumers who used food delivery services during the COVID-19 pandemic were more likely to have positive emotions because they were more easily able to access food than non-users [70].

**Figure 8:**
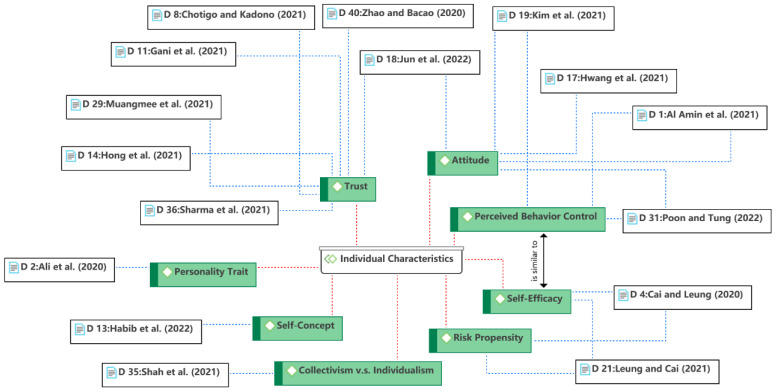
Theme 4: individual characteristics [6,12,23,26,35,36,37,39,40,43,46,50,52,56,58,59].

The fourth theme is the individual characteristics, as shown in Figure 8. In line with e-commerce literature, individual characteristics were also found to be a popular aspect influencing consumer behavior, with the most discussed being attitude and trust. Attitude is one of the core constructs of behavioral theories, such as the theory of reasoned action (TRA) [71] and the theory of planned behavior (TPB) [72]. Attitude as an individual characteristic has been broadly used in the e-commerce literature [73]. In general, consumers with positive attitudes toward information technology are more likely to use food delivery services. The role of trust has also been discussed in depth due to the uncertainty implicit in e-commerce [74,75,76]. In the context of food delivery, the trust may influence consumer perceptions of food quality or restaurant reputation (see Theme 8), which in turn affects the consumer decision-making process. TPB’s perceived behavioral control is similar to self-efficacy from social cognitive theory (SCT) [77], which were both viewed as individual characteristics in this theme, they are the extent to which individuals believe they can master a skill. During the pandemic, self-efficacy may refer to individuals’ belief that they can overcome COVID-19-related difficulties. Other individual characteristics discussed were culture (i.e., collectivism and individualism), risk propensity, personal traits (e.g., optimism), and sense of self, which may influence consumers’ use of food delivery services in different ways (directly or indirectly).

**Figure 9:**
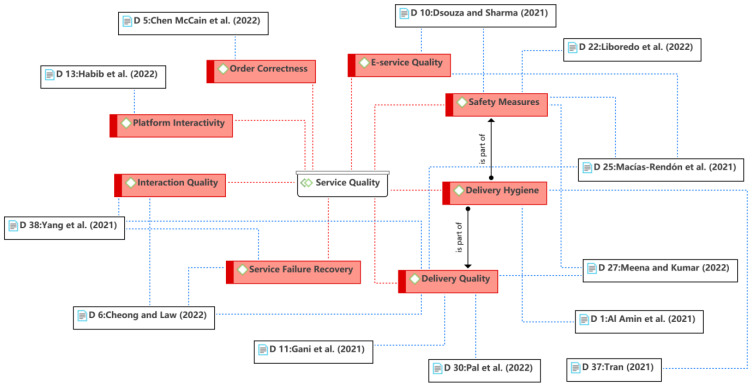
Theme 5: service quality [9,12,14,24,27,40,41,43,47,48,49,61].

The fifth theme is the service quality, as shown in Figure 9. In the traditional marketing literature, service quality was usually used as an antecedent of satisfaction to influence consumers’ purchase intention [78,79,80]. Similar to most O2O services, food delivery services involve both online and offline service quality [2]. Online service quality is achieved through e-service quality and platform interactivity, which are part of system service quality (see Theme 2). Offline service quality in the context of food delivery is mainly involved in delivery quality, including delivery time, order correctness, personal aspects of delivery workers, etc. Safety measures and hygiene issues of restaurants and delivery workers are new concerns of consumers caused by the COVID-19 pandemic. In addition, Cheong and Law [24] and Yang et al. [47] found that the interaction quality between restaurants and customers plays an influential role, especially during the pandemic. Such interaction can be online or offline, and the interaction with delivery workers is the most direct and most influential to the consumer’s perceived service quality. Macías-Rendón et al. [48] observed that consumers provide positive comments to delivery workers during the pandemic due to empathy (see Theme 7). Nevertheless, the literature showed that consumers may complain about delivery workers for a variety of reasons [81,82].

**Figure 10:**
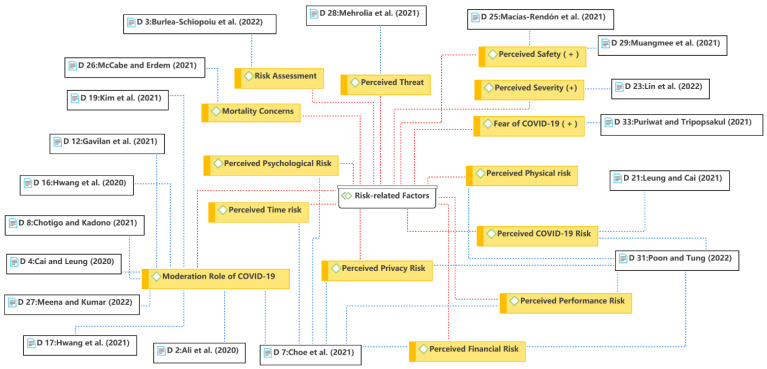
Theme 6: risk-related factors [9,13,15,23,26,28,34,35,37,38,39,44,48,52,54,56,59,60].

The sixth theme is the risk-related factors, as shown in Figure 10. As with trust discussed in Theme 5, perceived risk is widely studied in the e-commerce literature due to the uncertainty implied of the online environment. Risk-related factors formed the theme because of the increase in its discussion during the COVID-19 pandemic. Previous research has shown that perceived risk in the online environment involves many dimensions, such as performance risk, financial risk, time risk, privacy risk, and psychological risk [83]. In the reviewed studies, researchers focused on COVID-19-related risks in addition to discussing the traditional risk dimensions. Perceived risk usually negatively influences consumers’ behavioral intention or attitude; however, fear of COVID-19 [13] and perceived severity [44] were found to positively influence consumers’ intention to use food delivery services. This is because researchers viewed the perceived COVID-19-related risks from different perspectives (see Section 3.2.2). In addition, researchers focused more on COVID-19-related moderating effects, such as fear of COVID-19 [28], before and during the pandemic [35], severe and mild regions [26], and two COVID-19 waves [9]. COVID-19-related situations were observed to influence the consumer decision-making process in various ways.

**Figure 11:**
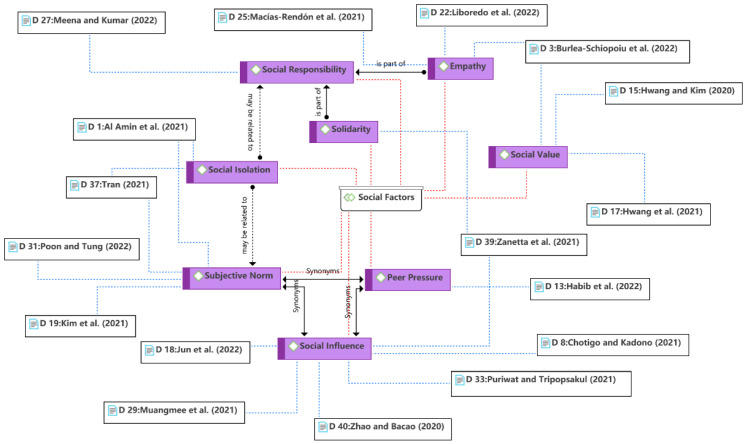
Theme 7: social factors [6,9,13,23,35,37,39,41,42,43,48,52,54,55,56,58,61].

The seventh theme is the social factors, as shown in Figure 11. Social factors are an essential aspect in both traditional behavioral theories (e.g., TRA and TPB) and technology use theories (e.g., UTAUUT). The subjective norm and social influence were found to be the most widely used constructs in the theme, which refer to the extent to which consumer behavior is influenced by others [66,67]. Consumer behavior in using food delivery services may be influenced by subjective norms or social pressures, as non-users (e.g., the elderly) may be socially excluded during the COVID-19 pandemic [84]. Social value is the perceived enhancement of the consumer’s self-concept or social prestige by using a certain food delivery service, such as using contactless food delivery during the pandemic. Consumer social responsibility is another aspect of social factors, which has been found to influence consumer behavior during the COVID-19 pandemic, mainly in the form of support or empathy for restaurants and delivery personnel affected by the crisis. Consumers’ complaints or other behaviors may make delivery workers’ livelihoods precarious [81,82]. However, during the pandemic, some consumers may increase their use of food delivery services or tip delivery workers due to social responsibility or empathy. In addition, social isolation was included in the theme as it relates to compliance with social norms or social responsibility regarding public policies during the pandemic.

**Figure 12:**
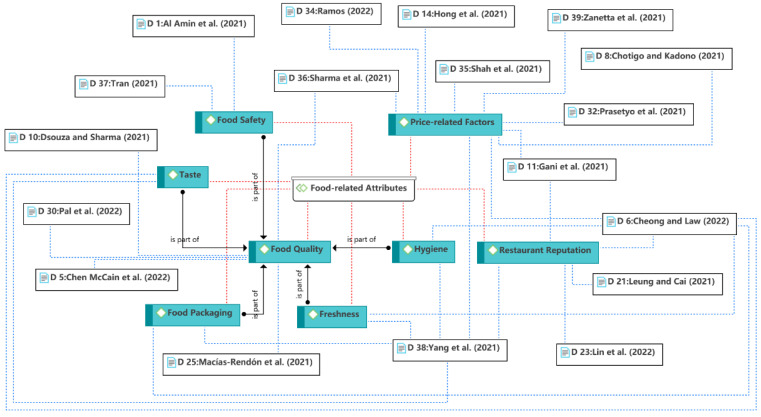
Theme 8: food-related attributes [12,14,24,27,35,36,40,42,44,46,47,48,49,50,51,53,59,61].

The eighth theme is the food-related attributes, as shown in Figure 12. The previous themes discussed why consumers use food delivery services. However, using the service is just a means for consumers to achieve their fundamental goals, namely, to purchase food. Previous studies have shown that food choice motives involve many aspects, such as health, taste, food quality, food safety, price, convenience, familiarity, etc. [21,85]. In this theme, price-related factors were the most discussed. No studies reported non-significant results for the price-related factors, except for Chotigo and Kadono [35], finding that the COVID-19 pandemic moderated the effect of price on satisfaction. Food quality, safety, and hygiene are also of concern to consumers and researchers during the pandemic. In addition, the restaurant reputation and taste aspects remain prominent in the customer experience [24,47]. However, health factors as an important aspect of food choice motives were not discussed in the reviewed articles. One possible explanation is that dietary health was not a major concern during the pandemic compared with fundamental food needs.

In addition to the eight themes identified, several codes that could not contribute to any themes nevertheless deserve attention, such as habit [35,42], use frequency [15,42,48], product involvement [15], consumer engagement [43], etc. Previous studies have demonstrated that habit is a key predictor of technology use [16,67]. Consumer involvement and online engagement mean more frequent use [86,87], which is related to habit formation. In fact, we are more interested in the antecedents of the habit. In other words, researchers should focus on what fosters or breaks consumers’ habits in the post-pandemic era.

In summary, this section addresses research question 2, generating eight themes pertinent to the factors influencing consumers’ use of food delivery services during the COVID-19 pandemic. The results are basically similar to the previous e-commerce literature [2,73], suggesting that these factors have been extensively discussed and examined in different literature. However, the elements and mechanisms of these themes may differ from those of normal times. The COVID-19-related elements are presented separately in Section 3.2.2.

#### 3.2.2. COVID-19-Related Themes

**Figure 13:**
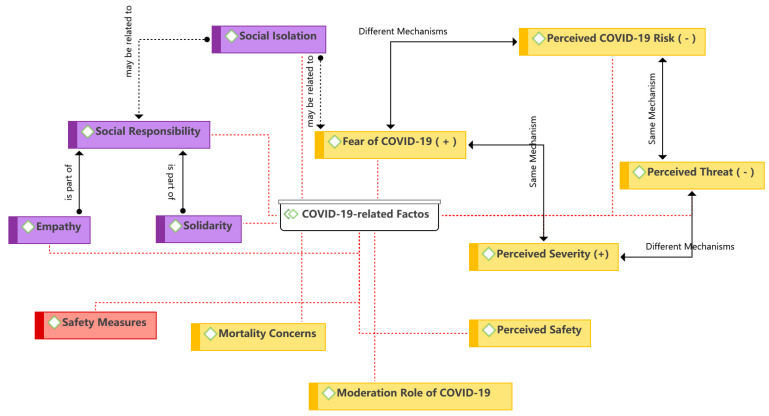
COVID-19-related factors.

Although conventional factors and theories can explain consumer behavior in using food delivery services during the pandemic, several new factors caused by COVID-19 have caused concern among researchers. Figure 13 presents an overview of the COVID-19-related factors. Among these, the mechanisms by which COVID-19-related risks influence consumer behavior were observed to be different. When the perceived COVID-19 risk is from online channels (i.e., delivery workers, food packaging, etc.), it negatively affects consumer behavior or behavioral intention to use food delivery services. Conversely, consumers are more likely to use online channels to purchase food if their fear is from offline channels (i.e., in-person offline purchases). Similarly, the roles of the perceived threat and perceived severity are different. Social responsibility is another topic of interest. A study found that corporate social responsibility is an important expectation of consumers during the pandemic [9]. Correspondingly, consumer social responsibility is one of the factors influencing their use of food delivery services, mainly expressed in terms of support and empathy for restaurants and delivery workers. In addition, social isolation was found to positively influence consumers’ use behavior or intention, possibly due to fear of COVID-19 or social responsibility to comply with public policies. Regarding safety measures, many restaurants or platforms implemented sanitization, contactless delivery, and other measures during the pandemic to respond to consumer concerns.

**Figure 14:**
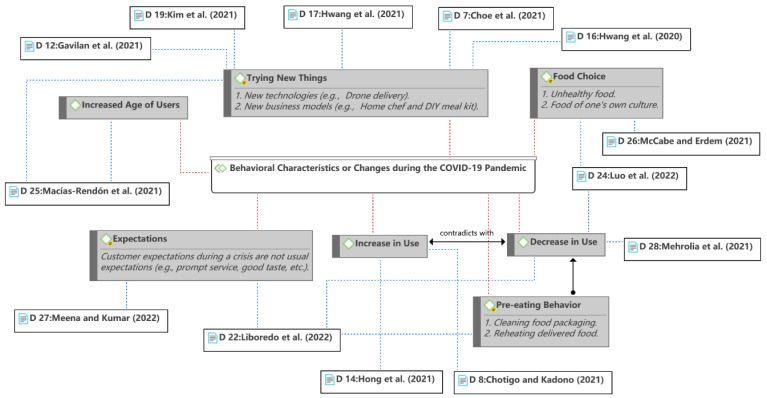
Observed behavioral characteristics or changes [9,15,28,34,35,36,37,38,39,41,45,48,60].

The COVID-19 pandemic has significantly changed people’s lifestyles and behaviors [23,24,39]. Several studies observed changes in consumer behavior in terms of using food delivery services during the pandemic, as shown in Figure 14. In line with most industry reports on food delivery services, Chotigo and Kadono [35] and Hong et al. [36] observed an increase in the frequency of use or number of new users. However, some studies observed a decrease in use [15,41,45]. One of the possible reasons for the inconsistency is the different mechanisms of consumers’ perceived COVID-19-related risks, as mentioned previously. Other reasons may come from differences in sample, culture, policy, etc. For example, students use fewer food delivery services when they study at home and live with their parents during the pandemic [45]; some users clean food packaging or reheat delivered food before consumption for various reasons [41]. Additionally, previous studies have shown that people aware of health risks may change their behavior in a preventive way [88,89,90]. This means that more new users may use the food delivery service, while existing ones may be more cautious.

During the COVID-19 pandemic, consumers were more concerned about safety measures and social responsibility than the usual expectations (e.g., prompt service and good taste). The pandemic may also influence consumers’ food choice preferences. Consumers may prefer food from their own culture because people counteract the psychological threat of death by supporting positive evaluations of their own cultural products [60]. On the other hand, the COVID-19 pandemic has had positive consequences in terms of technological advances and business model innovation. Consumers began to experiment with new technologies, such as drone delivery [39]; restaurants began to offer new services, such as Home Chef and DIY meal kits [28].

A study found that younger consumers are more likely to use food delivery services than older generations [36]. In fact, the respondents mentioned in most of the studies reviewed the young generations were predominant (i.e., Generation Y and Z). Nonetheless, the average age of users was observed to be significantly higher compared with that before the COVID-19 pandemic [48]. In addition, although an increase in the consumption of unhealthy foods was observed [45], the relationship between the use of food delivery services and unhealthy lifestyles during the pandemic has not been discussed.

To summarize, this section answers research question 3, partially presenting the influence of the COVID-19 pandemic on consumers in terms of using food delivery services.

### 3.3. Future Research Directions

As discussed in Section 1, it is necessary to continue studying consumer behavior in food delivery services in the post-pandemic era. Several future research directions are suggested based on our results.

#### 3.3.1. Dependent Variables or Outcome Phenomena

Firstly, since relatively few studies have focused on consumer behavior in the post- COVID-19 era, it is unclear whether consumers will continue to use food delivery services as frequently as they did during the pandemic. Consequently, consumers’ continued use intention or behavior can be one of the future research directions.

Secondly, overusing food delivery services may be associated with unhealthy lifestyles or negative outcomes. The use of food delivery services may contribute to a sedentary lifestyle, increasing the risk of adverse health outcomes [62]. Food safety and health issues have raised people’s concerns [91,92]; however, online food delivery platforms always seem to promote unhealthy food [93]. During the pandemic, people’s diets changed, especially as unhealthy diets increased [45,93,94]. Although Sharma et al. [50] investigated the over-ordering of food delivery service users, the discussion of the negative outcomes is not enough. Therefore, exploring the relationship between the use of food delivery services and unhealthy lifestyles or negative outcomes is suggested as a future research direction.

Thirdly, consumers’ green consumption behavior is another outcome phenomenon that needs to be concerned. Although green consumer behavior has long been discussed [95,96], it seems to be ignored in the food delivery industry, especially during the pandemic when people suffer from COVID-19. A large number of food delivery orders means massive amounts of packaging materials, typically non-biodegradable and challenging to recycle, leading to serious environmental problems [63]. Thus, future research should pay attention to green consumption issues in the food delivery industry.

#### 3.3.2. Independent Variables or Factors

The development of new habits or the disappearance of old ones depends on many aspects, such as individual social context changes, technological advances, public policies, natural disasters, etc. [25]. Future research can examine potential interventions to foster or break habits of using food delivery services. Furthermore, despite the efforts of restaurants and food delivery platforms to adopt various measures to encourage consumer purchases during the pandemic, the continued effectiveness of such marketing strategies is unclear. Maintaining these marketing efforts can challenge small and medium-sized restaurants or companies [14]. Therefore, it makes sense to continue studying the factors influencing consumer behavior (i.e., existing marketing strategies) in the post-pandemic era to develop new marketing strategies.

New factors are also worth being investigated. First, although consumer involvement and online engagement do not form any themes, they should continue to be studied. They mean more frequent use and may be related to habit formation. Second, consumer social responsibility is another future research direction. While existing studies have discussed the influences of consumer responsibility on the use of food delivery services during a pandemic, many other dimensions of consumer responsibility have received little attention, such as environmental responsibility.

#### 3.3.3. Research Contexts

The food delivery service is a broad category not only limited to ready-to-eat food delivery, which we can call O2O delivery service or to-home O2O business model. The COVID-19 pandemic has spawned many new business models, and more new ones will emerge as technology develops. For example, the concept of “food delivery” in China is constantly being broadened, with the rapid development of instant delivery services represented by fresh food and medicine [97]; drone delivery service in Korea is favored for its novelty and contactless delivery [39]; restaurants in Spain offer standard delivery services as well as experiential services such as Home Chef and DIY meal kits [28]. Future research can therefore focus on new research contexts, i.e., new business models.

In addition, the elderly suffered from increased social exclusion during the COVID-19 pandemic due to their inability to access food and necessities through food delivery platforms [84]. Technology acceptance and use among the elderly should be of concern to researchers. However, existing studies mainly investigated the younger generations of O2O delivery service users rather than the older ones. Therefore, future research can focus on the acceptance of technology or business models in the context of the elderly.

## 4. Conclusions

The popularity of e-commerce and mobile internet allows consumers to purchase food online through B2C or O2O models. The COVID-19 pandemic has accelerated the development of these business models, especially the to-home O2O, namely the food delivery service business. The provision or use of food delivery services is expected to become a new normal in the post-pandemic era. However, the discussion on consumer behavior toward food delivery services will continue. Therefore, the purpose of this paper was to review the literature on consumers’ use of food delivery services during the COVID-19 pandemic to offer a foundation and insights for future research.

A thematic review was conducted in this paper, with 40 articles published from 2020 to the present being reviewed. The quantitative results showed the current research trends and, to some extent, reflect the industry trends. The qualitative results mainly generated eight themes regarding the factors that influence consumer behavior in using food delivery services: (1) technical and utilitarian factors, (2) system-related attributes, (3) emotional and hedonic factors, (4) individual characteristics, (5) service quality, (6) risk-related factors, (7) social factors, and (8) food-related attributes. The influence of COVID-19 was subsequently highlighted. Based on the results, future research directions were suggested in three aspects: (1) dependent variables or outcome phenomena, (2) independent variables or factors, and (3) research contexts.

### 4.1. Contributions

This paper brings contributions in several aspects. First of all, this paper presents an overview to policymakers regarding consumer behavior in certain aspects in times of crisis. Meanwhile, this paper offers starting points for future research. It is comprehensive enough to help scholars understand how themes are formed and detailed enough to allow many different sub-themes to be focused on.

This paper also provides beneficial insights for marketers and managers in food-related industries. First, despite the similarity of the eight themes identified to previous marketing literature, their composition and the way they work may be different. Marketers and managers can gain a more comprehensive understanding of consumer behavior from this paper to reconsider their marketing strategies. Second, consumers may have different expectations in times of crisis than in normal times, for example, they may place more value on corporate social responsibility. Lastly, restaurants and food delivery platforms should manage human resources well in terms of delivery workers. Delivery is an essential part of the industry, and the delivery worker’s performance can directly influence the customer experience. Consumer behavior may, in turn, lead to precariousness among food delivery workers.

### 4.2. Limitations

A number of limitations need to be noted regarding this review paper. First, although we developed a detailed and comprehensive literature search strategy, it is possible to miss some valuable articles. Second, while the most difficult period of the COVID-19 pandemic has passed, relevant studies may be in the process of being published, resulting in not being included in this review. Lastly, the thematic review approach cannot examine the effectiveness of previous studies. Notwithstanding these limitations, this review benefits the industry practice and future research of O2O food delivery services.

## Figures and Tables

**Table 1:** Countries or regions of research by the year of publication.

No.	Countries or Regions	2020	2021	2022	Count *	References
1	Bangladesh		2		2	[12,40]
2	Brazil		1	1	2	[41,42]
3	China’s mainland	1	2	3	6	[6,43,44,45,46,47]
4	Ecuador		1		1	[48]
5	India		4	2	6	[7,9,14,15,49,50]
6	Indonesia		1		1	[51]
7	Macau			1	1	[24]
8	Malaysia			1	1	[52]
9	Mexico			1	1	[53]
10	Pakistan	1			1	[23]
11	Romania			1	1	[54]
12	South Korea	2	3		5	[34,37,38,39,55]
13	Spain		1		1	[28]
14	Thailand		3		3	[13,35,56]
15	Turkey		1		1	[57]
16	USA	1	3	3	7	[9,26,27,36,58,59,60]
17	Vietnam		1		1	[61]

* Some studies were cross-country

## Data Availability

Not applicable.

## Appendix A

**Table A1:** A list of articles included in the review.

No.	Articles	Journals
1	Al Amin et al. (2021) [40]	*Journal of Food Products Marketing*
2	Ali et al. (2020) [23]	*Journal of Open Innovation: Technology, Market, and Complexity*
3	Burlea-Schiopoiu et al. (2022) [54]	*Socio-Economic Planning Sciences*
4	Cai and Leung (2020) [26]	*International Journal of Hospitality Management*
5	Chen McCain et al. (2022) [27]	*British Food Journal*
6	Cheong and Law (2022) [24]	*International Journal of Environmental Research and Public Health*
7	Choe et al. (2021) [34]	*International Journal of Contemporary Hospitality Management*
8	Chotigo and Kadono (2021) [35]	*Sustainability*
9	Dirsehan and Cankat (2021) [57]	*Journal of Retailing and Consumer Services*
10	Dsouza and Sharma (2021) [14]	*International Journal of Innovation Science*
11	Gani et al. (2021) * [12]	*Journal of Foodservice Business Research*
12	Gavilan et al. (2021) [28]	*International Journal of Gastronomy and Food Science*
13	Habib et al. (2022) [43]	*Future Business Journal*
14	Hong et al. (2021) [36]	*Journal of Hospitality and Tourism Management*
15	Hwang and Kim (2020) [55]	*Sustainability*
16	Hwang et al. (2020) [38]	*International Journal of Environmental Research and Public Health*
17	Hwang et al. (2021) [37]	*Journal of Travel and Tourism Marketing*
18	Jun et al. (2022) [58]	*Foods*
19	Kim et al. (2021) [39]	*International Journal of Hospitality Management*
20	Kumar and Shah (2021) [7]	*Journal of Retailing and Consumer Services*
21	Leung and Cai (2021) [59]	*Journal of Hospitality and Tourism Management*
22	Liboredo et al. (2022) * [41]	*Nutrition* & *Food Science*
23	Lin et al. (2022) [44]	*Sustainability*
24	Luo et al. (2022) [45]	*European Journal of Nutrition*
25	Macías-Rendón et al. (2021) [48]	*Estudios Gerenciales*
26	McCabe and Erdem (2021) [60]	*Journal of Applied Social Psychology*
27	Meena and Kumar (2022) [9]	*Journal of Retailing and Consumer Services*
28	Mehrolia et al. (2021) [15]	*International Journal of Consumer Studies*
29	Muangmee et al. (2021) [56]	*Journal of Theoretical and Applied Electronic Commerce Research*
30	Pal et al. (2022) [49]	*Journal of Foodservice Business Research*
31	Poon and Tung (2022) * [52]	*European Journal of Management and Business Economics*
32	Prasetyo et al. (2021) [51]	*Journal of Open Innovation: Technology, Market, and Complexity*
33	Puriwat and Tripopsakul (2021) [13]	*Emerging Science Journal*
34	Ramos (2022) [53]	*British Food Journal*
35	Shah et al. (2021) * [46]	*British Food Journal*
36	Sharma et al. (2021) [50]	*International Journal of Hospitality Management*
37	Tran (2021) [61]	*Sustainability*
38	Yang et al. (2021) [47]	*International Journal of Hospitality Management*
39	Zanetta et al. (2021) [42]	*Food Research International*
40	Zhao and Bacao (2020) [6]	*International Journal of Hospitality Management*

* Article in press (early access).
